# Current News

**Published:** 1899-03

**Authors:** 


### Current News.

### OREGON STATE DENTAL ASSOCIATION.

THE officers of the Oregon State Dental Association for the ensuing year are as follows:

President, Dr. John Welch; First Vice-President, Dr. R. L. Lincoln; Second Vice-President, Dr. H. M. Hurd; Treasurer, Dr. C. E. Shalk; Secretary, Dr. Arthur W. Chance.

ARTHUR W. CHANCE,
*Secretary.*

